# Vital Signs: Evaluation of Hepatitis C Virus Infection Testing and Reporting — Eight U.S. Sites, 2005–2011

**Published:** 2013-05-10

**Authors:** Katherine Bornschlegel, Deborah Holtzman, R. Monina Klevens, John W. Ward

**Affiliations:** New York City Dept of Health and Mental Hygiene, New York, New York; Div of Viral Hepatitis, National Center for HIV/AIDS, Viral Hepatitis, STD, and TB Prevention, CDC

## Abstract

**Background:**

Hepatitis C virus (HCV) infection is a serious public health problem. New infections continue to occur, and morbidity and mortality are increasing among an estimated 2.7–3.9 million persons in the United States living with HCV infection. Most persons are unaware of their infection status. Existing CDC guidelines for laboratory testing and reporting of antibody to HCV do not distinguish between past infection that has resolved and current infection that requires care and evaluation for treatment. To identify current infection, a test for HCV RNA is needed.

**Methods:**

Surveillance data reported to CDC from eight U.S. sites during 2005–2011 were analyzed to determine the proportion of persons newly reported on the basis of a positive test result for HCV infection. Persons reported with a positive result from an HCV antibody test only were compared with persons reported with a positive result for HCV RNA and examined by birth cohort (1945–1965 compared with all other years), surveillance site, and number of reported deaths. Annual rates of persons newly reported with HCV infection in 2011 also were calculated for each site.

**Results:**

Of 217,755 persons newly reported, 107,209 (49.2%) were HCV antibody positive only, and 110,546 (50.8%) were reported with a positive HCV RNA result that confirmed current HCV infection. In both groups, persons were most likely to have been born during 1945–1965 (58.5% of those who were HCV antibody positive only; 67.2% of those who were HCV RNA positive). Among all persons newly reported for whom death data were available, 6,734 (3.4%) were known to have died; deaths were most likely among persons aged 50–59 years. In 2011, across all sites, the annual rate of persons newly reported with HCV infection (positive HCV antibody only and HCV RNA positive) was 84.7 per 100,000 population.

**Conclusions:**

Hepatitis C is a commonly reported disease predominantly affecting persons born during 1945–1965, with deaths more frequent among persons of relatively young age. The lack of an HCV RNA test for approximately one half of persons newly reported suggests that testing and reporting must improve to detect all persons with current infection.

**Implications for Public Health:**

In an era of continued HCV transmission and expanding options for curative antiviral therapies, surveillance that identifies current HCV infection can help assess the need for services and link persons with infection to appropriate care and treatment.

## Introduction

In the United States, hepatitis C virus (HCV) infection is a common bloodborne infection. Based on data from national surveys, an estimated 3.2 (95% confidence interval [CI] = 2.7–3.9) million persons in the United States are living with hepatitis C (1). Once infected, approximately 80% of persons remain infected (i.e., chronically infected) and are at risk for substantial morbidity and mortality in later life (2). Although treatment can be curative, an estimated 45%–85% of infected persons are unaware of their HCV infection (3). HCV infection is a major cause of liver disease, including cirrhosis and liver cancer (4–7), and in the United States, is the leading indication for liver transplantation (8). Moreover, rates of liver cancer and deaths from HCV infection have increased over time; approximately 15,000 HCV-associated deaths were recorded in 2007 (4,9). In addition, considerable costs are associated with HCV infection, both in lost productivity and health-care expenditures (10–11).

CDC guidelines for HCV laboratory testing and reporting, published in 2003, do not focus on identifying persons with current infection (12); therefore, depending on the HCV test used, reports to surveillance programs can include persons with a test result indicating past HCV infection that has resolved and also persons with a test result that identifies current HCV infection. Analysis of state and local surveillance data can be used to assess the proportion of persons who might need additional testing to discriminate previous resolved infection from current infection. Analysis of such data also can estimate the number of persons with current HCV infection requiring clinical assessment for treatment, as well as guide prevention strategies. In addition, these surveillance data can serve as a baseline for indirectly evaluating use of the recent HCV testing recommendations to identify HCV infection among persons born during 1945–1965, a group that demonstrates the highest prevalence of infection, compared with those born in other years (3). Finally, examining mortality patterns among persons reported with current HCV infection can improve understanding of the natural history of the disease.

## Methods

In 2011, CDC supported surveillance for HCV infection at eight U.S. sites (Colorado, Connecticut, Minnesota, New Mexico, New York City, New York state, Oregon, and San Francisco). CDC began receiving data in 2005 from four sites (Colorado, Minnesota, New York state and Oregon), one site in 2006 (New Mexico), two sites in 2008 (New York City and San Francisco), and one site in 2009 (Connecticut). For all sites, clinical laboratories reported only positive test results of HCV infection (i.e., from HCV antibody testing or from HCV RNA testing); health departments did not require reporting of negative results. Reports were reviewed and de-duplicated to ensure that persons with newly reported positive HCV test results were included only once in the surveillance database.

For this analysis, persons reported to CDC during 2005–2011 were categorized as 1) reported with only a positive test result for HCV antibody (HCV antibody positive only) or 2) reported with a positive HCV RNA result from HCV nucleic acid testing or HCV genotyping (HCV RNA positive). Persons who tested HCV antibody positive only were considered as having had a past HCV infection that had resolved, a false-positive test result, or current HCV infection. Persons who tested HCV RNA positive were considered currently HCV infected. Although no laboratory test exists to distinguish acute from chronic HCV infection, for the purpose of this study all persons determined to be currently infected were considered to have chronic infection.

Each group (HCV antibody positive only and HCV RNA positive) was examined by birth cohort (1945–1965 compared with all other birth years) and surveillance site. Annual rates of all persons newly reported per 100,000 population in 2011 also were calculated for each site using denominators available from U.S. Census population estimates (available at http://www.census.gov/compendia/statab). In addition, seven of the sites reported the frequency of known deaths from any cause among persons newly reported with HCV infection. Sites matched their hepatitis C databases with vital records at the person level. Death status was examined by sex, age group, birth cohort, and type of test result (HCV antibody positive only or HCV RNA positive).

## Results

During 2005–2011, among the eight sites, a total of 217,755 persons were newly reported with a positive test result for HCV infection. Of these, 107,209 (49.2%) were HCV antibody positive only and 110,546 (50.8%) were HCV RNA positive. In both groups, persons were more likely born during 1945–1965. Persons born during these years accounted for 58.5% of those who were HCV antibody positive only and 67.2% of those who were HCV RNA positive (Table 1). The distribution of persons reported on the basis of positive HCV antibody only varied by site, ranging from 76% in New Mexico to 23% in Minnesota (Figure). Among sites reporting deaths, 6,734 (3.4%) of 197,844 persons newly reported with HCV infection were known to have died. The highest percentage of these deaths occurred among persons aged 50–59 years (44.8%), and most deaths (71.5%) were among those born during 1945–1965, compared with other years. The percentage of deaths among persons reported with HCV antibody positive only (4.6%) was significantly higher than among those reported as HCV RNA positive (2.4%; p<0.01). In 2011, the annual rate of all persons newly reported with HCV infection (positive HCV antibody only and HCV RNA positive) across all sites was 84.7 per 100,000 population (range: 36.0 in Minnesota to 239.2 in San Francisco) (Table 2).

## Conclusions and Comment

These data show that approximately one half of persons newly reported with HCV infection to state or local authorities at eight surveillance sites did not have a report of a positive HCV RNA test; thus, it was not possible to determine whether the reports indicated past resolved HCV infection or current HCV infection. Previous studies have shown similar results. A separate analysis of surveillance data reported for 2006–2007 found that 47.3% of persons reported with positive HCV antibody did not have HCV RNA test results (13). A multisite cohort study of patients in care for chronic viral hepatitis revealed that 37.7% of 9,086 patients with a positive HCV antibody test during 2006–2008 had no documented follow-up testing for HCV RNA (14). A retrospective study of HCV antibody testing in selected U.S. primary-care settings among persons born during 1945–1965 found that, among patients who were antibody positive, 32% received no follow-up HCV RNA testing (15). In New York City, 33% of persons reported through routine surveillance did not have HCV RNA testing (16).

Given these findings and recent developments in both HCV testing technologies and clinical care for persons with HCV infection, CDC is amending the guidelines for HCV laboratory testing and result reporting that have been in use since 2003 (12). In guidance accompanying this Vital Signs report, CDC recommends following a positive HCV antibody test with HCV RNA testing (17). This guidance is also consistent with that provided in the 2012 HCV testing recommendations for persons born during 1945–1965 (3). The new guidelines will help identify persons with current HCV infection and provide the data necessary to link those who are infected to care, including preventive services, medical management, and evaluation for antiviral treatment.

An unexpected result was the finding of a significantly greater percentage of deaths among persons who were HCV antibody positive only compared with those who were HCV RNA positive. Because persons in the latter group have demonstrated current infection, they would be expected to fare less well than those who were HCV antibody positive only and might or might not be currently infected. The difference between the groups in the percentage of deaths might be explained by health-care access. HCV RNA testing might not be available in sites providing HCV antibody testing and RNA testing requires successful referral to a health-care provider. Thus, this finding could suggest that persons reported on the basis of a positive HCV antibody test only might have had less opportunity to access health care or might have accessed health care less often than those with current infection.

This study also revealed a high rate of reported HCV infection at these U.S. sites, especially among persons born during 1945–1965. These findings reinforce recent CDC recommendations for HCV antibody testing of persons born during 1945–1965, and linkage to care for those with a follow-up positive result after HCV RNA testing (3). These data further showed that deaths were more likely among persons aged 50–59 years and among persons born during 1945–1965 compared with those born in other years, illustrating the important impact of HCV infection on years of life lost.

**Key Points**

- CDC guidelines for laboratory testing and result reporting of antibody to hepatitis C virus (HCV) published in 2003 and developed in the era of limited treatment options fail to identify many persons with current HCV infection. As such, about one half of persons newly reported with hepatitis C lack HCV RNA results, which are necessary to identify current infection.
- In 2011, the overall annual rate of persons newly reported with hepatitis C was 84.7 per 100,000 population; rates varied by site.
- The highest percentage of persons with current HCV infection and the highest percentage of deaths among all persons newly reported with hepatitis C were among those born during 1945–1965, particularly those aged 50–59 years.
- Additional information is available at http://www.cdc.gov/vitalsigns.

The findings in this report are subject to at least five limitations. First, state and local health departments only report positive HCV test results to CDC. Thus, it was not known whether persons who were reported HCV antibody positive only might actually have been tested for HCV RNA with a negative result. Another possibility is that HCV RNA testing was performed with a positive result, but was not reported. Second, some positive HCV antibody test results might have been false-positives. However, the high specificity of 3rd generation HCV antibody assays used during the period of study would have minimized the number of false positives (18). Third, among sites, there was variation in reporting by health-care providers, laboratories, and health departments, which might affect the consistency of the information reported. For example, the Connecticut hepatitis C surveillance system did not enter HCV RNA results for persons reported with a positive antibody test that previously had been confirmed to be positive for antibody to HCV by another laboratory test. Fourth, some sites began reporting surveillance data to CDC in 2006 or 2008, and in one case, 2009, thereby underestimating the number of cases reported during the entire 2005–2011 study period. In contrast, the number of deaths reported was from all-cause mortality, and therefore was likely an overestimation of HCV-attributable mortality. Finally, HCV surveillance data might not be representative of all persons with HCV infection, and the findings from these eight sites might not be representative of other U.S. cities and states.

Monitoring current HCV infection in states and localities can help gauge what interventions and services are needed to identify persons with HCV infection and effectively link them to appropriate care and treatment. This is of particular importance now in an era of continued HCV transmission and rapidly improving therapeutic options for persons living with HCV infection. To help identify persons with current HCV infection, public health and clinical care providers can offer HCV antibody testing to persons born during 1945–1965, in addition to those with other HCV risk factors, and test for HCV RNA those persons who test positive for HCV antibody. Laboratories can ensure that test results are reported to state and local health authorities, and health departments can develop strategies to monitor and increase the use of HCV RNA testing of persons who are HCV antibody positive.

## Figures and Tables

**FIGURE:**
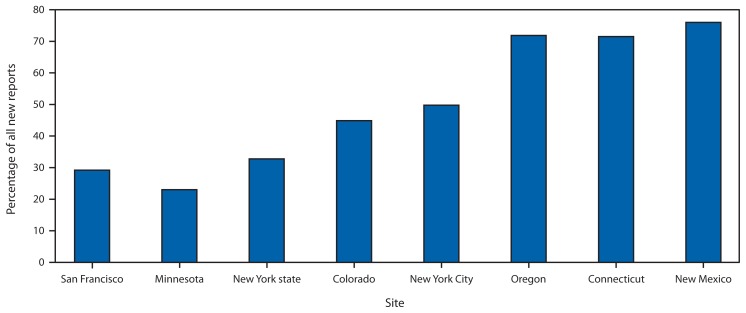
Percentage of persons newly reported with a positive result from a hepatitis C virus (HCV) antibody test only among all new reports with positive HCV test results, by site — eight U.S. sites, 2005–2011

**TABLE 1:** Percentage of persons newly reported with positive test results for hepatitis C virus (HCV) infection, by birth cohort and type of test result — eight U.S. sites, 2005–2011

	HCV antibody positive only		HCV RNA positive		Total	
			
Birth cohort	No.	(%)	No.	(%)	No.	(%)
Born during 1945–1965	62,728	(58.5)	74,270	(67.2)	**136,998**	**(62.9)**
Born in other years	44,481	(41.5)	36,276	(32.8)	**80,757**	**(37.1)**
**Total**	**107,209**	**(100.0)**	**110,546**	**(100.0)**	**217,755**	**(100.0)**

**TABLE 2:** Number and rate per 100,000 population of persons newly reported with positive test results for hepatitis C virus (HCV) infection (HCV antibody positive only or HCV RNA positive), by site — eight U.S. sites, 2011

Site	No.	Site population	Rate per 100,000
Colorado	2,901	5,116,796	56.7
New Mexico	3,188	2,082,224	153.1
San Francisco	1,944	812,826	239.2
Minnesota	1,925	5,344,861	36.0
New York state	7,047	11,220,287	62.8
Oregon	5,464	3,871,859	141.1
Connecticut	2,898	3,580,709	80.9
New York City	8,749	8,244,910	106.1
**Total**	**33,919**	**40,274,472**	**84.7**
